# Determination of Palatal Soft Tissue Thickness and Safe Zone for Palatal Soft Tissue Harvest Using CBCT: A Retrospective Study

**DOI:** 10.1155/2023/8417073

**Published:** 2023-06-12

**Authors:** Hanan Aldhanhani, Bhavna Jha Kukreja, Sesha Reddy, Jovita D'souza, Hossam Abdelmagyd

**Affiliations:** ^1^Department of Preventive Dental Sciences, College of Dentistry, Gulf Medical University, Ajman, UAE; ^2^Department of Oral Medicine and Periodontology, Faculty of Dentistry, Suez Canal University, Ismailia, Egypt

## Abstract

**Aim:**

Our decision to conduct this study was motivated by the dearth of knowledge on geographical variations in the thickness of the palatal masticatory mucosa. The aim of the present study is to comprehensively analyze the palatal mucosal thickness and indicate the safety zone for palatal soft tissue harvesting using cone beam computed tomography (CBCT). *Material and Methods*. As this was a retrospective analysis of cases previously reported to the hospital, written consent was not acquired. The analysis was carried out on 30 CBCT images. Two examiners evaluated the images separately to avoid bias. Measurements were done from the midportion of the cementoenamel junction (CEJ) to the midpalatal suture in a horizontal line. Measurements were recorded from the maxillary canine, first premolar, second premolar, first molar, and second molar and were marked in axial and coronal sections at distances of 3, 6, and 9 mm from the CEJ. The relationship between palate soft tissue thickness in relation to each tooth, palatal vault angle, teeth, and the greater palatine grove was evaluated. Differences in the palatal mucosal thickness according to age, gender, and tooth site were evaluated. Categorical data were presented as frequencies and percentages. Numerical data are presented as mean and standard deviation values. They are explored for normality using Shapiro–Wilk's test. Data are normally distributed and are analyzed using one-way ANOVA followed by Tukey's post hoc test for independent variables and paired *t*-test for repeated measures. The significance level is set at *p* ≤ 0.05 for all tests. Statistical analysis is performed with R statistical analysis software version 4.1.3 for Windows.

**Results:**

For sex and nationality, there was no significant association (*p* > 0.05), while for age, cases 35 years and older had significantly higher mucosal thickness than cases younger than 35 years old (*p* < 0.001). For all teeth, the association was statistically significant (*p* < 0.001). For the canine and first premolar, cases with deep angles had significantly higher mean values than those with moderate angles (*p* < 0.001). For other teeth, cases with deep angles had significantly higher mean values than other angles (*p* < 0.001).

**Conclusion:**

Palatal mucosal thickness varied significantly from the canine to the second molar; the most appropriate site for graft harvesting is the canine to second premolar area which is 9–12 mm from the midpalatal suture aspect and is considered a safe zone for harvesting palatal graft.

## 1. Introduction

Regardless of the patient's dental hygiene, periodontal and peri-implant recessions are multifactorial conditions that will worsen periodontium. Midbuccal recessions were reported by more than 50% of an adult population sample, according to earlier research [1]. According to the US National Survey, a recession is present at one or more sites for 88% of senior citizens (age 65 and above) and 50% of persons (18–64); as people become older, recessions are shown to occur more frequently and to a greater extent. The incidence of the recession was 37.8% and the magnitude was 8.6% teeth in the middle age group (30–39 years). The frequency was 90.4% (twice as high) and the average number of teeth was 56.3% (over six times as many) in the oldest cohort, which was made up of people who were 80–90 years old [2, 3].

In terms of effective management for the gingival recession, we have both nonsurgical and surgical methods. The term “periodontal plastic procedures” refers to a group of surgical methods used to enhance what is now known as “the pink esthetic.” These treatments aim to provide excellent outcomes while also striking a balance between function and aesthetics [4]. Numerous surgical procedures have been developed over time to treat labial gingival recession abnormalities. Despite various surgical procedures available to treat gingival recession, subepithelial connective tissue grafts (SECT) are still considered the gold standard operating procedure in the management of a different class of gingival recessions [5].

The most popular donor sites for SECT and free gingival grafts are the maxillary tuberosity and lateral palate. Owing to the substantial tissue thickness to keratinized gingiva ratio, the region between the first bicuspid and second molar is regarded as the most ideal harvesting location. However, there might be differences in gingival thickness and histologic makeup between people, and even within the same patient [6–9]. The maximum volume of soft tissues that have been harvested, in terms of height and length, have previously been documented, together with variances in essential structures and palatal vault morphology [10].

It is advised to assess the palatal mucosal thickness (PMT) before the surgical procedures since it might have a direct impact on how the surgery will be planned [11]. Due to different anatomical characteristics, soft tissue transplantation from the palate should be done with extra caution to avoid any significant consequences. Any harm to the neurovascular bundle in this area might result in consequences including paresthesia and bleeding. To acquire the maximal dimensions of the graft from the proper location, it is crucial to validate the thickness of the mucosa before beginning the surgical treatment. This may be accomplished using a variety of methods. Bone sounding or transgingival probing is the most well-known and established of these methods. This method's obvious drawback is that anesthesia is needed. Additional methods for evaluating PMT include the use of ultrasonography, magnetic resonance imaging (MRI), or cone-beam computed tomography [12–14].

Finding ultrasound probes in oral-friendly sizes and performing accurate equipment calibration are the challenges when utilizing ultrasonography [15]. The software that has been created for MRI allows for digital 3D modeling, and PMT measurement has been proven to be accurate [16]. The imaging method known as MRI is noninvasive and radiation-free, but it also has negative effects, including a high price, a lengthy scanning duration, and claustrophobia for the patients [17]. Cone beam computed tomography (CBCT) is widely used to quantify palatal mucosal thickness because it gives researchers information with a high degree of diagnostic quality and allows for millimeter-level analysis. Recently it was revealed that CBCT can also be utilized for imaging dentogingival soft tissues with studies completed in recent years, even though extensive evaluation of hard tissues in the craniofacial complex rather than soft tissues is advised for CBCT in the early years [18, 19].

The bundle of palatal neurovascular tissue is the most significant anatomical structure in the palate. The pterygopalatine fossa and pterygopalatine canal are both traversed by the neurovascular bundle, which travels along and contains the palatal artery, vein, and nerve. Given all of this data, a CBCT scan can provide essential information to assist and ensure that the neurovascular bundle is not damaged and to obtain the proper quantity of graft when planning procedures that require soft tissue grafting from the palate. To the best of our knowledge, Arab or Mediterranean ethnic patients were not considered in any of the earlier noninvasive investigations using CBCT. Our decision to conduct this study was motivated by the dearth of knowledge on geographical variations in the thickness of the palatal masticatory mucosa. The aim of the present study is to comprehensively analyze the palatal mucosal thickness and indicate the safety zone for palatal soft tissue harvesting using CBCT.

## 2. Materials and Methods

The study was conducted at the University Dental Hospital, UAE, from May 2021 to April 2022 for a duration of 12 months. The study was approved by the Institutional Review Board of Gulf Medical University (ref. no. IRB/COD/STD/50/Apr-2021).

### 2.1. Sample Selection

CBCT images were taken from the oral radiology department. Images taken for either implant surgery or orthodontic treatment purposes were selected. The inclusion criteria included the bilateral presence of teeth from the second molar to canine and scans that were taken with a byte to create enough space to view the soft tissue contrast. The striking effect of metallic restorations, missing teeth, tongue occluding the surrounding soft tissue, local pathology, severe crowding, rotation, and spacing in the maxilla was excluded. As this was a retrospective analysis of cases previously reported to the hospital, written consent was not acquired. The analysis was carried out on 30 CBCT images. Two examiners evaluated the images separately to avoid bias.

### 2.2. Radiographic Measurement of Palatal Soft Tissue Thickness

**Figure 1:**
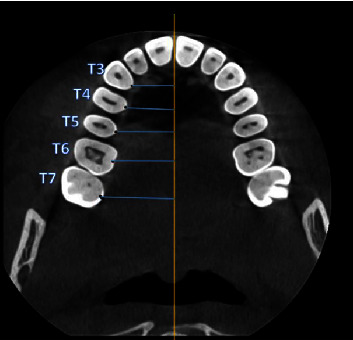
CBCT-axial view shows the definition of measurement points. Each point was marked from the midpalatal of the cementoenamel junction (CEJ) to the middle palatine suture (blue line) and the yellow line resembles the middle palatine suture.

**Figure 2:**
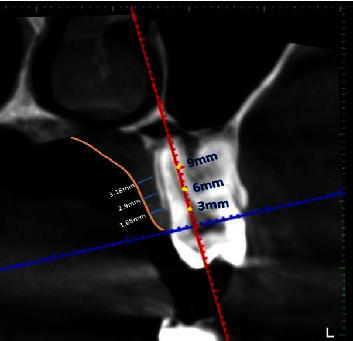
CBCT-coronal view shows each point that was marked from the cementoenamel junction with a 3 mm interval (yellow dots) along a line bisecting the palatal root as a reference. A perpendicular line (blue line) joins these points to the tangential line drown (pink line).

ProMax3DMid (Planmeca) was used to obtain CBCT images. Image acquisition's technical specifications were 90 kVp, 8 mA, 12 s, voxel size 0.2 mm, and slice thickness of 600 mm. Measurements were done from the midportion of the cementoenamel junction (CEJ) to the midpalatal suture in a horizontal line. Measurements were recorded from the maxillary canine, first premolar, second premolar, first molar, and second molar and were marked in axial and coronal sections at distances of 3, 6, and 9 mm from the CEJ. In the coronal view each point that was marked from the CEJ with a 3 mm interval (yellow dots) along a line bisecting the palatal root as a reference. A perpendicular line (blue line) joins these points to the tangential line drawn (pink line) (Figure 1). In the coronal view, each point that was marked from the CEJ with a 3 mm interval (yellow dots) along a line bisecting the palatal root as a reference. A perpendicular line (blue line) joins this point to the tangential line drawn (pink line) (Figure 2).

**Figure 3:**
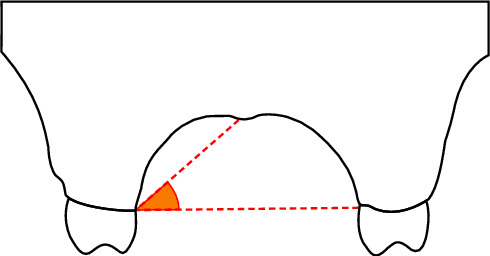
The palatal vault angle on the maxillary first molar was measured using the junction angle between the horizontal plane at the cementoenamel junction (CEJ) and an imaginary line from the midpalatal suture to the CEJ.

**Figure 4:**
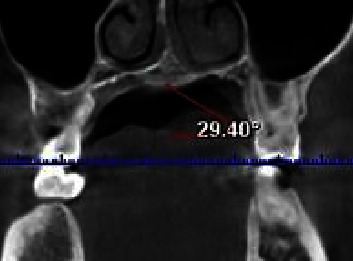
Showing a shallow palate with palatal vault angle <30°.

**Figure 5:**
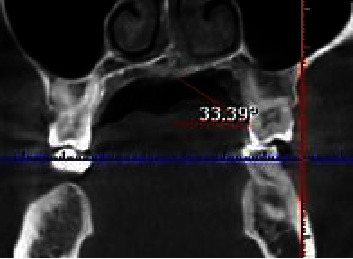
Showing moderate palatal with palatal vault angle 30°–40°.

**Figure 6:**
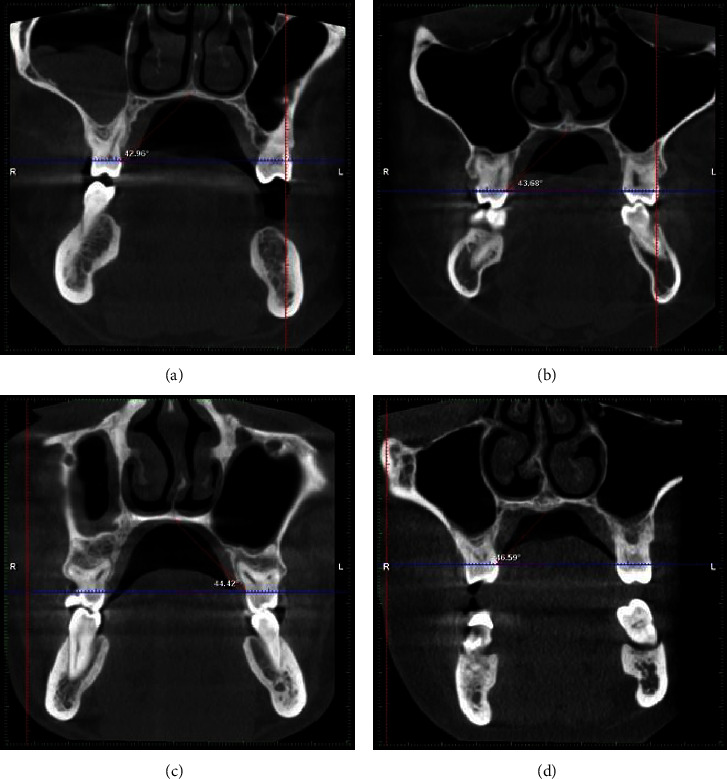
(a–d) Deep palate with palatal vault angle >40°.

In coronal pictures, the angle between the horizontal plane at the CEJ and a line drawn from the midpalatal suture was used to estimate the palatal vault angle on the maxillary first molar (Figure 3). The images were divided into three groups based on the palatal vault angle on specific sections of the maxillary first molar: shallow group (Group S) with the angle being less than 30° (Figure 4), moderate group (Group M) with the angle being between 30° and 40° (Figure 5), and deep group (Group D) with the angle being more than 40° (Figure 6).

**Figure 7:**
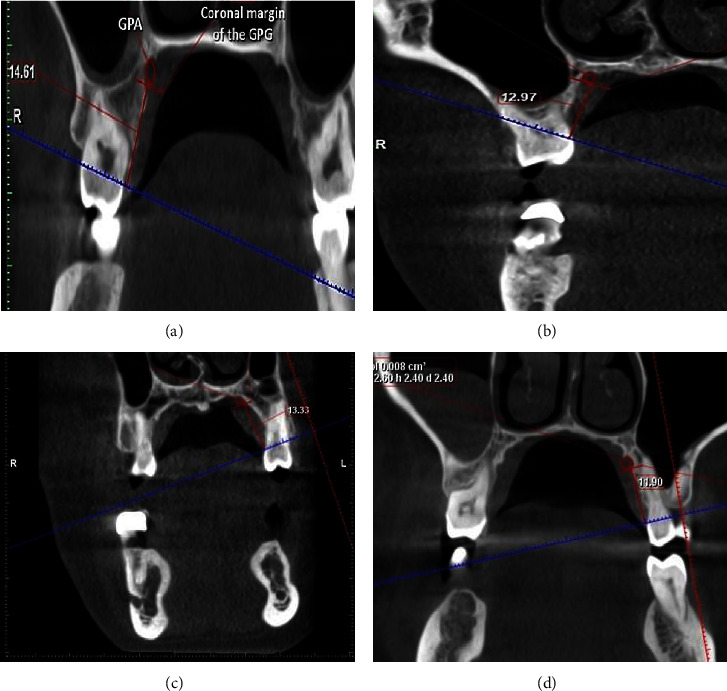
In coronal sections, (a) and (b) show the distance in mm from midpalatal CEJ of the molar to the coronal margin of the greater palatine groove. (c) and (d) Demonstrate the distance between the greater palatine groove's coronal margin and the premolar's midpalatal CEJ.

The greater palatine foramen (GPF) and palatine groove (PG) locations were assessed to gauge the surgical risk associated with tissue harvesting by measuring the distance between the midpoint of the palatal cementoenamel junction of each tooth (T3–T7) to the corresponding greater PG (Figure 7).

### 2.3. Statistical Analysis

A power analysis was designed to have adequate power to apply a statistical test of the null hypothesis that there is no difference between tested groups. By adopting an alpha (*α*) level of (0.05), a beta (*β*) of (0.2) (i.e., power = 80%), and effect size (*f*) of (0.637) calculated based on the results of a previous study [20], the minimum required sample size (*n*) was found to be (27) cases. Sample size calculation was performed using G^*∗*^Power version 3.1.9.7. Analysis was carried out on CBCT images of 30 subjects (17 males; 16 females; mean age 32).

Categorical data were presented as frequencies and percentages. Numerical data are presented as mean and standard deviation values. They are explored for normality using Shapiro–Wilk's test. Data are normally distributed and analyzed using one-way ANOVA followed by Tukey's post hoc test for independent variables and paired *t*-test for repeated measures. The significance level is set at *p* ≤ 0.05 for all tests. Statistical analysis is performed with R statistical analysis software version 4.1.3 for Windows.

## 3. Results

### 3.1. Characteristics of Demographics

The association between demographic data and mucosal thickness is presented in Table 1. For sex and nationality, there was no significant association (*p* > 0.05). While for age, cases 35 years and older had significantly higher mucosal thickness than cases younger than 35 years old (*p* < 0.001) (Table 1).

### 3.2. Palatal Soft Tissue Thickness with Regard to Each Tooth

The association between measurement distance and mucosal thickness is presented in Table 2 and Figure 2. For all teeth, the association was statistically significant (*p* < 0.001). For the canine, the value measured at 6 mm was significantly higher than values measured at other distances (*p* < 0.001). For the first premolar, the value measured at 3 mm was significantly lower than values measured at other distances (*p* < 0.001). For the second premolar, values measured at different distances significantly differed (*p* < 0.001). For the first and second molars, the value measured at 9 mm was significantly higher than values measured at other distances (*p* < 0.001) (Table 2).

### 3.3. Correlations between Palatal Vault Angle and Thickness of Palatal Soft Tissue

The association between the palatal vault angle and the palatal mucosal thickness is presented in Table 3 and Figures 4 and 5. For different teeth, there was no significant association between palatal vault angle and mucosal thickness (*p* > 0.05).

### 3.4. Correlations between Palatal Vault Angle and Greater Palatine Groove Course

A comparison between teeth regarding the greater PG course is presented in Table 4 and Figure 6. There was a significant difference between different teeth (*p* < 0.001), with the second molar having a significantly higher value than other teeth (*p* < 0.001), the first molar and second premolar having significantly higher values than the first premolar and canine (*p* < 0.001), and with the first premolar having a significantly higher value than the canine (*p* < 0.001).

### 3.5. Association between Teeth and Greater Palatine Groove Course

For all teeth, the association was statistically significant (*p* < 0.05). The association between measurement distance and mucosal thickness is presented in Table 5 and Figure 7. For the canine and first premolar, cases with deep angles had significantly higher mean values than those with moderate angles (*p* < 0.001). For other teeth, cases with deep angles had significantly higher mean values than other angles (*p* < 0.001).

## 4. Discussion

To our knowledge, only two studies have been published on the reliability of radiographic techniques for determining palatal mucosal thickness and palatal vaults [20–23]. When doing soft tissue grafting in periodontal plastic surgery, the palatal masticatory mucosa serves as the primary donor location for connective tissue. The thickness of the graft is directly related to the surgical success of soft tissue transplants. The shortcomings of invasive techniques such as volume being affected by anesthesia, inflammation, and the anatomical structure of the palatal region along with repeated measurement for ultrasound technique thus drove researchers to look for an alternative to existing methods to measure the PMT. When compared with traditional tomography, CBCT offers several benefits, including less radiation exposure, improved picture quality, increased patient comfort, and cheaper cost [24].

CBCT was therefore utilized in the current retrospective investigation to gauge the thickness of the palatal mucosa. The palatal mucosa thickness differed between age groups in the current investigation. The average thickness of the palatal mucosa was 2.8 mm in people under 35 years and 3.11 mm in people over 35 years. The current investigation shows that the palatal mucosa thickness increases with aging, which is consistent with other studies. This is due to excess deposition of adipose tissue, mucous glands, high prevalence of exostoses, and gingival recession thereby resulting in palatal mucosal thickening. Also, this could be explained by the fact that the hard palate mucosa is an orthokeratinized epithelial layer that thickens with age. Aging is also associated with changes in gingival tissue, which is known to become coarser and denser [12, 13, 19].

Furthermore, there was no significant relationship between gender, ethnicity, and palatal gingival thickness in our study. However, females showed a slightly higher mean thickness (3.04 mm) than males (2.93 mm). The findings of our study were in contrast with previous researchers who reported thin mucosal tissue in females than their counterparts [9, 13] but were in line with other scholars [12, 25].

Arabs had a mean thickness of 2.98 mm and non-Arabs had 2.9 mm. The mean thickness of palatal mucosa in our study was less than that of the previous research which could be attributed to race [26]. These contradictory results might have been caused by a variety of variables, including variations in the respondents' ethnic backgrounds, reference structures, and assessment methodologies. This calls for more research using bigger sample sizes. The palate mucosal thickness may also be influenced by other confounding variables such as race, genetics, and body weight. To date, only one study has been done on the Arab population which was an invasive method by bone sounding [26].

From the canine to the second premolar molar region, the palatal mucosa's overall thickness increased, and this increased thickness was accompanied by a rise in thickness from the CEJ apically or at 3, 6, and 9 mm onto the root surface. The canine–premolar region is the best location for graft harvesting because the upper first molar region has the thinnest mucosa in the hard palate, which can be explained by the position and curve of the palatal root of the maxillary first molar. The results of the recent study agreed with those of the earlier researchers. Age, ethnicity, different measuring techniques, and the positioning of the measurement sites might all play a role in this variation [12, 19, 26].

The greater palatine artery (GPA) and the greater palatine nerve, which emerge from the GPF, travel anteriorly over the palate in a groove, and terminate at the incisive canal, are other factors that affect the graft size [27]. The donor site that is best suited for palatal tissue graft harvest corresponds to the area where the vascular-nerve bundle emerges from the larger palatine foramen. Typically, that bundle is defined as being 10–14 mm from the gingival margin, distal to the third molar, or between the third and second molars [28].

In the present study, there was a significant difference between different teeth with the second molar having a significantly higher value than other teeth regarding the greater PG course. Distance between the GPA and the maxillary canine teeth reduces as it travels anteriorly over the palate, with maximum distance being noticed for the second molar. Similar findings were reported in the past regarding the distance between the greater palatine course and the gingival margin of teeth [29]. Having knowledge of these neurovascular bundles is of utmost importance during harvesting soft tissue graft as it contains greater palatine artery, vein, and nerve. These neurovascular bundles are typically 7, 12, and 17 mm from the CEJs of the premolars and molars, respectively, depending on the height of the palatal vault. Due to comparable tissue densities, CBCT cannot detect greater palatine bundles in soft tissue; nonetheless, CBCT-assisted diagnosis and anatomical understanding might lessen surgical problems.

Only recently a few studies have been done to investigate the associations between PMT and palatal vault angle using CBCT images, and this could be one among them. Based on the angle formed by the horizontal plane at the CEJ and a line drawn from the midpalatal suture, the palatal vault angle on the maxillary first molar was determined in coronal images. Images were split into three groups based on the palatal vault angle. Furthermore, it has been demonstrated that the GPA's position correlates with the form of the palatal vault. The GPA is closer to the cementoenamel junction in shallow palates. The graft width that may be obtained depends on how far the GPA is from the CEJ. The posterior extent of the incision is determined by the GPF, which is often situated in the second or third molar area.

The findings of the current study are that there is no association between palatal vault angle and mucosal thickness. This was similar to previous results in spite of the difference in palatal vault angle measurement [19] but refuted by other studies [13, 30].

A statistical analysis found no correlation between a moderate, deep, or shallow palate and PMT, in our study. The different approaches taken to measuring the palatal depth might be the cause of the discrepancy in the results. Observations of this study present that there are no statistical differences between the degree of the palatal vault and the thickness of the palatal masticatory mucosa that supports the idea that individuals with a steep palatal vault angle have surgical difficulties in harvesting palatal grafts.

## 5. Conclusion

Anatomical features and tissue thickness may restrict the amount of palatal graft that may be harvested. According to this study palatal vault angle does not affect the mucosal thickness but supports the fact that neurovascular bundles emerging from the greater palatine course determine the graft dimension. A safe zone to harvest palatal grafts lies between the canine and the second premolar region. The graft harvest is unaffected by smoking, gender, the side of the mouth, or gingival phenotype. When working with patients who have shallow palatal vaults, the operating surgeon must be cautious since the donor graft may not be enough in terms of dimensions. The possible dangers of taking soft tissue transplants from deeper into the palate must be investigated in more detail.

## Figures and Tables

**Table 1:** Association between demographic data and mucosal thickness.

Parameter	Mean ± SD	*p*-value
Sex		
Male	2.93 ± 0.89	0.069
Female	3.04 ± 0.94	
Age group		
Younger than 35 years old	2.88 ± 0.88	<0.001^*∗*^
35 years and older	3.11 ± 0.94	
Nationality		
Arab	2.98 ± 0.91	0.822
Non-Arab	2.99 ± 0.92	

^*∗*^Statistically significant.

**Table 2:** Association between the measurement distance and mucosal thickness for different teeth.

Tooth	Mucosal thickness (mean ± SD)			
	3 mm	6 mm	9 mm	*p*-value
Canine	3.07 ± 0.80^B^	3.43 ± 0.80^A^	2.89 ± 0.74^B^	<0.001^*∗*^
First premolar	2.66 ± 0.66^B^	3.51 ± 0.74^A^	3.81 ± 0.76^A^	<0.001^*∗*^
Second premolar	2.49 ± 0.60^C^	3.35 ± 0.59^B^	3.95 ± 0.74^A^	<0.001^*∗*^
First molar	2.21 ± 0.63^B^	2.44 ± 0.63^B^	3.11 ± 0.56^A^	<0.001^*∗*^
Second molar	2.24 ± 0.90^B^	2.38 ± 0.78^B^	3.18 ± 0.99^A^	<0.001^*∗*^

^*∗*^Statistically significant. ^A,B,C^Different superscripts letters within the same horizontal row are significantly different.

**Table 3:** Association between palatal vault angle and mucosal thickness.

Tooth	Mucosal thickness (mean ± SD)			*p*-value
	Shallow	Moderate	Deep	
Canine	3.14 ± 0.83^A^	3.09 ± 0.91^A^	3.16 ± 0.70^A^	0.856
First premolar	3.12 ± 0.86^A^	3.31 ± 0.89^A^	3.39 ± 0.86^A^	0.515
Second premolar	3.03 ± 0.79^A^	3.22 ± 0.95^A^	3.35 ± 0.83^A^	0.352
First molar	2.61 ± 0.84^A^	2.52 ± 0.75^A^	2.65 ± 0.66^A^	0.466
Second molar	2.93 ± 0.95^A^	2.71 ± 1.13^A^	2.43 ± 0.80^A^	0.082

^A^Different superscripts letters within the same horizontal row are significantly different.

**Table 4:** Comparison between teeth regarding the greater palatine groove course.

Distance of the greater palatine groove (mean ± SD)					*p*-value
Canine	First premolar	Second premolar	First molar	Second molar	
9.24 ± 1.07^D^	10.46 ± 1.12^C^	12.24 ± 1.28^B^	12.15 ± 1.06^B^	13.08 ± 1.28^A^	<0.001^*∗*^

^*∗*^Statistically significant. ^A,B,C,D^Different superscripts letters within the same horizontal row are significantly different.

**Table 5:** Association between palatal vault angle and greater palatine groove course.

Tooth	Distance of the greater palatine groove (mean ± SD)			*p*-value
	Shallow	Moderate	Deep	
Canine	8.96 ± 0.73^AB^	8.80 ± 0.88^B^	9.71 ± 1.12^A^	0.005^*∗*^
First premolar	10.27 ± 1.08^AB^	10.05 ± 1.06^B^	10.88 ± 1.07^A^	0.018^*∗*^
Second premolar	11.23 ± 0.76^B^	11.68 ± 0.91^B^	12.97 ± 1.28^A^	<0.001^*∗*^
First molar	11.38 ± 0.56^B^	11.72 ± 0.86^B^	12.70 ± 1.04^A^	<0.001^*∗*^
Second molar	12.23 ± 0.21^B^	12.46 ± 0.93^B^	13.83 ± 1.29^A^	<0.001^*∗*^

^*∗*^Statistically significant. ^A,B^Different superscripts letters within the same horizontal row are significantly different.

## Data Availability

A retrospective analysis was carried out on CBCT images of 30 subjects who visited Thumbay Dental Hospital Ajman which have been recorded by the HIMS system.
